# Effective atomic numbers and electron density of dosimetric material

**DOI:** 10.4103/0971-6203.54853

**Published:** 2009

**Authors:** S. B. Kaginelli, T. Rajeshwari, Sharanabasappa, B. R. Kerur, Anil S. Kumar

**Affiliations:** Department of Physics, Gulbarga University, Gulbarga - 585 106, India; 1 Radiation Safety and System Division, Bhabha Atomic Research Centre, Mumbai - 400 085, India

**Keywords:** Mass attenuation coefficient, effective atomic number, electron density, dosimetric materials

## Abstract

A novel method for determination of mass attenuation coefficient of x-rays employing NaI (Tl) detector system and radioactive sources is described.in this paper. A rigid geometry arrangement and gating of the spectrometer at FWHM position and selection of absorber foils are all done following detailed investigation, to minimize the effect of small angle scattering and multiple scattering on the mass attenuation coefficient, μ/ρ, value. Firstly, for standardization purposes the mass attenuation coefficients of elemental foils such as Aluminum, Copper, Molybdenum, Tantalum and Lead are measured and then, this method is utilized for dosimetric interested material (sulfates). The experimental mass attenuation coefficient values are compared with the theoretical values to find good agreement between the theory and experiment within one to two per cent. The effective atomic numbers of the biological substitute material are calculated by sum rule and from the graph. The electron density of dosimetric material is calculated using the effective atomic number. The study has discussed in detail the attenuation coefficient, effective atomic number and electron density of dosimetric material/biological substitutes.

## Introduction

In medical physics it is important to evaluate the amount of radiation, delivered by the ionizing radiation, in composite substances. The energy delivered through the photon interactions in composite substances cannot represent the atomic number uniquely across the entire energy region. This number in composite substances is called the effective atomic number and it varies with energy and is denoted here by Z_eff_. On the other hand, the concept of z-dependence of photon attenuation coefficient has been utilized in many applications of radiation studies.[1] For example, precise knowledge of effective atomic numbers is very important in medical radiation dosimetry and medical imaging, where the cross-sectional anatomy is generated by computer tomography (CT) scans.[2] It is a common practice to verify the validity of calculation algorithms by comparing the generated doses with the measured doses in tissue equivalent phantom substances. Similarly, tissue-equivalent phantoms are specifically designed to study the image quality and performance of the CT scanners. In both instances, a precise knowledge of the effective atomic number and electron density of the composite substances is necessary in the low energy region and have proved to be a convenient parameter for interpretation of x-ray attenuation by a complex medium like a biological tissue and particularly in the calculation of dose in radiography and radiation dosimetry etc.[3]

The importance of this paper from diagnostic or therapeutic point of view is that while calculating the effective atomic number of the compound, especially when the photon energy is close to the binding energy of the electron present in the compound, it gives correct information about corrections to be added while calculating the dose to the patient. In such cases the experimental determined effective atomic number may not be agreeable with the theoretical values i.e. deviates from calculated values using the Jackson's formula.

The x-ray mass attenuation coefficient, μ/ρ, for any material is usually estimated from Bragg's additivity law or more commonly called mixture rule. Thus μ/ρ for any chemical compound/material is given by

μ/ρ=∑ωi(μ/ρ)i,

where (μ/ρ)_i_ is the mass attenuation coefficient of the i^th^ element and ω_i_ is the fraction by weight of the i^th^ element. For a compound/material with chemical formula (Z_1_)_a1_, (Z_2_)_a2_,……(Z_n_)_an_ the weight factor for the i^th^ element is given by

ωi={(aiAi/(∑aiAi)},

where A_i_ is the atomic weight of the i^th^ element. Hence an attempt has been made, in this regard, to determine the μ/ρ of x-rays for the dosimetric material (sulfates of Mg, Ca, Mn Fe and Zn elements) and then determine the Z_eff_ of these material by LSF method from ln(μ/ρ) Vs. lnZ graph. These values are compared with the theoretical values. Using the these values of Z_eff_ the effective electron density calculated by the expression

Ne=NAZeff/Aeff

Where *N*_A_ is the Avogdro's number, A_eff_ = *A/n_i_* is the effective atomic weight is the ratio of the molecular weight of the sample divided by the total number of the atoms of all types present in the compound.

## Experimental

**Figure 1:**
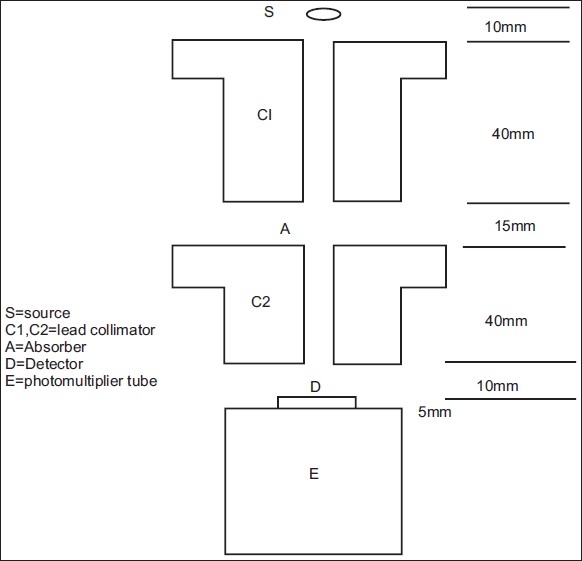
Experimental arrangement

The good-geometry experimental arrangement used in determination of the mass attenuation coefficient is similar to the one described in detail by us earlier[4] and the schematic experimental setup is shown in Figure 1. Briefly, photons from a variable energy x-ray source S passed through a collimator C1 and were incident on the specimen A in the form of a thin foil/pellet kept normal to the photon beam. The transmitted beam passed through another collimator C2 and reached a NaI (Tl) x-ray detector D. The transmitted photon spectrum was recorded using a PC-based multi-channel analyzer.

The collimators C1 and C2 were 40 mm thick lead discs that collimated respectively the incident and transmitted beam to 6 mm dia. The scatter acceptance angle equal to the sum of the incident beam divergence and acceptance angle at the detector is found to be less than or equal to 3° degrees. This thickness of the collimator would reduce the intensity of scattered photons of 300 keV by a factor of 10^7^.

A radioactive isotope ^57^Co of strength 0.37 MBq (10 μCi), was obtained from BRIT, Mumbai, India. ^57^Co decays by electron capture to the ^57^Fe ground state, emitting Fe K_1_, K_α2_ and K_β_ x-rays. The NaI(Tl) detector can't resolve K_α_ and K_β_ x-rays. In the present paper it is proposed that the mass attenuation coefficients are to be determined for the K_α_ x-ray only and removing the K_β_ x-rays. Hence the K_β_ x-ray intensity has practically been eliminated by the differential absorption technique. So, the transmitted beam was regarded to have the weighted average energy of K_α1_ and K_α2_ i.e. K_α_ is 6.400 keV. Similarly, for the ^65^Zn radioactive source only 8.907 keV x-rays selected for the final measurement of mass attenuation coefficient. The variable energy x-ray source consisted of 10 mCi (370 MBq) ^241^Am as the primary source of excitation radiation and Rubidium target selected to produce fluorescence x-ray with characteristic energy of the target ie., 13.339 keV. The inner brems-strahlung intensity was found to be negligible compared to the x-ray intensity in the region of interest from the radioactive sources. No noticeable impurities were observed in the source spectra.

The dosimetric compounds viz., MgSO_4_.7H_2_O, CaSO_4_.2H_2_O, MnSO_4_.H_2_O, FeSO_4_.7H_2_O, ZnSO_4_.7H_2_O of 99.5% purity were obtained from SD fine chemicals Mumbai, India.. Metal foil (Al, Cu, Mo and Ta) standards were procured from Good Fellow, England was used for standardization purposes only. The dosimetric samples of required thickness in the range of 10 to 200 mg/cm^2^ were prepared in the form of 10 mm dia cylindrical pellets by pressing the weighed quantity of the finely ground powder in a hand operated hydraulic press at a pressure of 10 ton. The area density (mass per unit area) of a foil/pellet sample was determined by weighing it using a single pan electronic balance with an accuracy of 0.01 mg and measuring its dimensions using a traveling microscope with an accuracy of 0.001 cm. Thus, the measured areal density expressed in mg/cm^2^ had an uncertainty of less than one per cent.

A bicron-made integrated assembly of 25mm dia × 4 mm thick Na(Tl) scintillation mounted on a photomultiplier tube (PMT) served as the x-ray detector. Oxford model PCAP plus PC plug-in single PCI card had on board high voltage supply, pre-amplifier, amplifier, and 1k ADC. The components on the card were individually controlled and used as 1k channel MCA using the software package OXWIN MCA.

The error involved in each measurement is taken care of by following the procedure counting time conditions as stated in Rose and Shapiro,[5] viz., background to signal background to foil thickness and signal to foil thickness, systematic errors due to the detection of forward scattered radiation, beam hardening when higher atomic number absorber is used. The Ray-sum method has been adopted in the present measurement for calculation for the random errors which arises from all aspects of the measurement, further is has also suggested a method for the calculation called the ray-sum error. In the present measurements, Ray-sum method is applied to all observations since the random errors arise from all aspects of measurement, in the exponential law of attenuation. The errors presented in the tables are due to propagation of errors calculated according the formula given by Pearson and Osborne.[6]

No dead time corrections are found for radioactive ^57^Co and ^65^Zn sources. However, in the present case we have selected the live time of the MCA for sources. With these conditions, the transmitted intensity of x-rays for various combinations of specimen thickness is recorded and corrected for background intensity.[7] A plot of logarithm of transmission as a function of specimen thickness yielded a straight line for the entire transmission region, verifying the validity of the Beer-Lambert's law. This is confirmed for different material too.

## Results and Discussion

**Figure 2:**
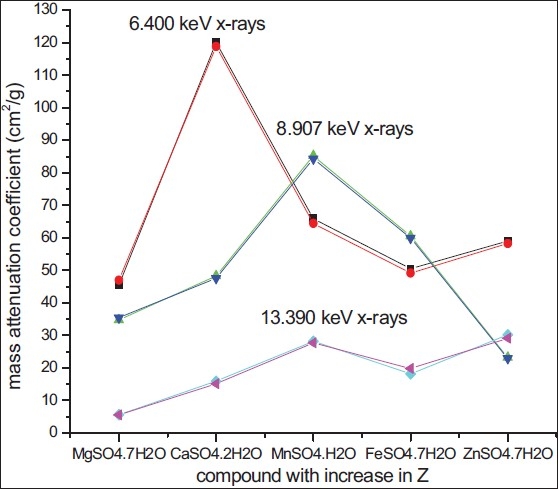
Mass attenuation coefficient Versus dosimetric compound with increase in Z

The plots of the logarithm of transmitted intensity versus specimen thickness were linear for all the samples and the μ/ρ is obtained from the plots of linear regression over the 50-2% transmission range. The μ/ρ obtained for the all dosimetric compounds at three different photon energies are presented in Table 1. The theoretical results have been calculated by WinXCom[8] or its predecessor, XCOM[9] using the mixture rule and theoretical μ/ρ values of the elements. The theoretical estimated errors are lying between one to two per cent as mentioned in the WinXCom.[8] The error involved in over all experimental values is lying between 2-3% for the dosimetric samples. The experimental μ/ρ values are presented in second column and theoretical μ/ρ are presented in the third column of Table 1. The percent deviation is the difference between the experimental and theoretical μ/ρ values divided by theoretical value. The determined values of μ/ρ in this transmission range is agreeing well with the theoretical values of WinXCom[8] within two to three per cent. Figure 2 clearly shows that the edge effect on the mass attenuation coefficient values otherwise all the three lines would have shown liner graph with a positive slope. As energy increases, μ/ρ decreases, which is well known fact that μ/ρ strongly depends on the atomic number of the absorber and inversely proportional to the energy.

**Table 1:** Experimental and theoretical mass attenuation coefficient for dosimetric material

*Name of the Sample*	*Mass attenuation coefficient (cm^2^/g)*		*P D in %*
	
	*Expt. value*	*WinXcom*	
	6.400 keV		
MgSO_4_.7H_2_O	45.5±0.6	46.99	-3.17
CaSO_4_.2H_2_O	120.0±1.2	118.8	1.70
MnSO_4_.H_2_O	65.9±0.9	64.4	2.33
FeSO_4_.7H_2_O	50.5±0.5	49.1	2.85
ZnSO_4_.7H_2_O	59.0±0.6	58.2	1.37
	8.907 keV		
MgSO_4_.7H_2_O	34.7±0.6	35.4	-1.98
CaSO_4_.2H_2_O	48.3±0.8	47.6	1.47
MnSO_4_.H_2_O	85.2±0.9	84.3	1.06
FeSO_4_.7H_2_O	60.5±0.8	59.9	1.00
ZnSO_4_.7H_2_O	23.2±0.4	22.9	1.31
	13.339 keV		
MgSO_4_.7H_2_O	5.62±0.06	5.53	1.63
CaSO_4_.2H_2_O	15.9±0.2	15.1	5.30
MnSO_4_.H_2_O	28.2±0.3	27.7	1.80
FeSO_4_.7H_2_O	18.2±0.2	19.8	8.08
ZnSO_4_.7H_2_O	30.2±0.3	29.1	3.78

PD = Percent difference =[(Experimental mean μ/ρ- Computed μ/ρ) / Computed μ/ρ] × 100

The extrapolated effective atomic number values of the dosimetric material are presented in Table 2 and these values found to vary from 10.25 to 18.43 for all the sulfates. The theoretical values of the Z_eff_ are also calculated using the formula given by Jackson and Hawkes[2] and these values are discussed in the light of the dosimetry point of view and as discussed in the introduction. The experimental and theoretical Z_eff_ values are agreeing within 5% except the edge region. It is important to mention that the theoretical/calculated values have not considered the edge effects and since the effective atomic numbers are under/over estimated when any element falls below the absorption edge. In the present work, there is a good indication that even in the low photon energy region say that up to 15 keV the effective atomic number can be determined with greater accuracy but one should take into account of edge effects. The electron density of the dosimetric materials is calculated using the experimental Z_eff_ values and found to vary 0.478 to 0.676 (10^24^ electrons g^−1^).

**Table 2:** Experimental and theoretical effective atomic number and electron density for dosimetric material

*Name of the sample*	*Effective atomic number (Z_eff_)*		*Electron density N_e_ (10^24^ electrons g^−1^)*

	*Expt*	*Jackson1981*	
	6.400 keV		
MgSO_4_.7H_2_O	7.21	7.65	0.543
CaSO_4_.2H_2_O	10.25	9.88	0.676
MnSO_4_.H_2_O	14.01	15.08	0.588
FeSO_4_.7H_2_O	11.18	19.33	0.478
ZnSO_4_.7H_2_O	10.48	16.26	0.613
	8.907 keV		
MgSO_4_.7H_2_O	10.07	9.88	0.644
CaSO_4_.2H_2_O	13.76	15.08	0.577
MnSO_4_.H_2_O	15.99	19.33	0.513
FeSO_4_.7H_2_O	14.42	16.26	0.843
ZnSO_4_.7H_2_O	10.87	20.98	0.614
	13.339 keV		
MgSO_4_.7H_2_O	10.33	9.88	0.681
CaSO_4_.2H_2_O	14.02	15.08	0.588
MnSO_4_.H_2_O	18.65	19.33	0.598
FeSO_4_.7H_2_O	15.89	16.26	0.929
ZnSO_4_.7H_2_O	18.43	20.98	1.042

## Conclusions

The experimental and theoretical Z_eff_ values are agreeing within five per cent except at the edge region and the determined Z_eff_ value is agreeing with the theoretical values within five per cent for three energies mentioned.
